# Crystal structures of *Escherichia coli* glucokinase acetylation-mimicking variants and insights into the impact of acetylation

**DOI:** 10.1107/S2053230X26002803

**Published:** 2026-05-01

**Authors:** Joseph Andrews, Joshua Sakon, Chenguang Fan

**Affiliations:** a Department of Chemistry and Biochemistry University of Arkansas, Fayetteville Fayetteville AR72701 USA; b Cell and Molecular Biology Program University of Arkansas, Fayetteville Fayetteville AR72701 USA; Centre for Cellular and Molecular Biology, Hyderabad, India

**Keywords:** glucokinases, lysine acetylation, acetylation-mimicking mutations

## Abstract

In this study, we report the crystal structures of K214Q and K216Q variants of *Escherichia coli* glucokinase (ecGLK), each of which is bound to phosphate in the active-site cleft. The structure of the K214Q variant was determined at 2.70 Å resolution and refined with an *R*_work_ and *R*_free_ of 0.140 and 0.190, respectively, while that of the K216Q variant was determined at 2.44 Å resolution with an *R*_work_ and *R*_free_ of 0.178 and 0.225, respectively. Both variants adopt an open conformation and maintain phosphate-binding interactions similar to the wild-type ecGLK. Structural comparison of the K214Q variant revealed large backbone deviations in the 214–224 α-helix, increased disorder in the loops surrounding the glucose-binding cleft and outward shifts of Asn99, Asp100, His160 and Glu187. Our previous study demonstrated that lysine acetylation at Lys214 and Lys216 impaired the activity of ecGLK, and here we show that acetylation mimics produced domain shifts, indicating those of lysine residues that could be essential for stabilizing the glucose-binding region of ecGLK.

## Abstract 2

This work reports the crystal structures of K214Q and K216Q variants of *E. coli* glucokinase, demonstrating a potential role of lysine acetylation in regulating glucokinase activity.

## 1. Introduction

The first step of the glycolytic pathway is catalyzed by glucokinase (GLK) and is a regulatory target in many organisms. GLKs are grouped into three distinct families according to their sequences. Group I enzymes are found in several eukaryotes and archaea; they are ADP- or ATP-dependent (Sakuraba *et al.*, 2004 ▸; Ronimus & Morgan, 2004 ▸). Group II enzymes include *Escherichia coli* glucokinase (ecGLK) and are ATP-dependent enzymes that lack the classic repressor open reading frame kinase (ROK) motif (Titgemeyer *et al.*, 1994 ▸; Meyer *et al.*, 1997 ▸). Group III enzymes originate from archaea and bacteria and are ATP-dependent enzymes that have the ROK sequence motif (Hansen *et al.*, 2002 ▸; Hansen & Schönheit, 2003 ▸).

Structural studies by Lunin and coworkers characterized ecGLK in the apo state and the glucose-bound complex (Lunin *et al.*, 2004 ▸). Crystallization of the apo state yielded crystals belonging to space group *P*4_3_2_1_2, with unit-cell dimensions *a* = *b* = 81.5, *c* = 234.7 Å. Crystallization of the glucose complex produced crystals belonging to space group *P*12_1_1, with unit-cell dimensions *a* = 78.416, *b* = 53.538, *c* = 90.903 Å. On superimposing the apo state and the glucose-bound complex, the maximum C^α^ deviation in the small domain was observed at Thr78, indicating that the portion of the loop containing Thr78 closes upon glucose binding. In the apo state, the loop region was characterized as intrinsically flexible. It was proposed that the flexible loop of ecGLK is stabilized upon glucose binding. This work highlighted the conformational transition required for glucose binding from the apo state to the glucose-bound complex.

Recently, our group solved structures of phosphate-bound ecGLK with and without bound glucose (Andrews *et al.*, 2025 ▸). The phosphate-bound ecGLK crystals had unit-cell dimensions *a* = *b* = 82.1, *c* = 237.9 Å in space group *P*4_3_2_1_2, and the crystals of the phosphate-bound glucose–ecGLK complex had unit-cell dimensions *a* = 47.5, *b* = 66.3, *c* = 207.5 Å in space group *P*2_1_2_1_2_1_. Structural characterization revealed a phosphate-binding site adjacent to the glucose-binding site, which interacts with His160, His183, Ser185, Arg188 and Arg286. A reduced *B* factor resulting from phosphate interactions within the α4, α5 and α9 helices suggested that the open state is more stable in these regions. The structural comparison located flexible regions before β1 and inside the β5–β6 loop, consistent with the flexibility and mobility observed before closure induced by glucose. In the phosphate-bound glucose–ecGLK complex, phosphate remains bound adjacent to glucose, and additional *B*-factor reduction in α4, α5 and α9 indicated that phosphate binding stabilizes the regions associated with closure.

Our previous enzyme kinetic study on ecGLK demonstrated that acetylation of Lys214 or Lys216 impaired the activity of ecGLK by affecting substrate binding, with approximately tenfold increases in the *K*_m_ values for both glucose and ATP, while the *k*_cat_ values were not significantly influenced (Fatema *et al.*, 2024 ▸). Interestingly, both Lys214 and Lys216 are not located in the active site. To further investigate the role of lysine acetylation at these two sites, in this work we solved the crystal structures of the ecGLK acetylation-mimicking variants K214Q and K216Q. Structural comparison of the K214Q variant revealed large backbone deviations in the 214–224 α-helix, increased disorder in the loops surrounding the glucose-binding cleft and outward shifts of Asn99, Asp100, His160 and Glu187, implying a possible role in stabilizing the glucose-binding region.

## 2. Materials and methods

### 2.1. Purification and crystallization

Expression and purification of ecGLK were performed as described in our previous structural study (Andrews *et al.*, 2025 ▸). The *glk* gene from *E. coli* was cloned into pCDFDuet-1 with a C-terminal His_6_-tag and transformed into *E. coli* BL21 (DE3) cells. The Lys-to-Gln mutations were introduced using the Q5 site-directed mutagenesis kit. Expression strains were grown in 400 ml LB medium containing 100 µg ml^−1^ streptomycin at 37°C until the absorbance reached 0.6–0.8 at 600 nm. Expression of ecGLK was induced by the addition of 0.1 m*M* isopropyl β-d-1-thiogalactopyranoside (IPTG) and incubation with shaking for a further 12 h at 16°C. The cells were then harvested by centrifugation for 20 min at 3000*g*. EcGLK purification started with the the addition of 12 ml lysis buffer (Tris–HCl pH 7.5, 300 m*M* NaCl, 20 m*M* imidazole) to the pellet tube with 5 µl β-mercaptoethanol, nuclease and protease inhibitors. The mixture was then sonicated on ice and centrifuged for 25 min at 19 000*g*. The supernatant was extracted and filtered through a 0.45 µm filter. The filtered supernatant was added to an affinity-chromatography column containing 2 ml Ni–NTA resin equilibrated with 20 ml lysis buffer. The column was then washed with 15 ml washing buffer (Tris–HCl pH 7.5, 300 m*M* NaCl, 50 m*M* imidazole) and 2.5 ml elution fractions were collected using elution buffer (Tris–HCl pH 7.5, 300 m*M* NaCl, 200 m*M* imidazole). The elution fractions were loaded onto a PD-10 column for desalting with desalting buffer (5 m*M* Na_2_HPO_4_, 1 m*M* NaH_2_PO_4_, 10 m*M* NaCl, 1 m*M* DTT). The protein sample was concentrated by centrifugation using a protein concentrator with a 10 kDa cutoff, yielding a protein concentration of 10 mg ml^−1^. Rigaku Berkeley Screen solutions 1–96 were used for crystal screening. Sitting-drop vapor diffusion was used with two drops, one containing 0.9 µl protein solution and 0.9 µl well solution and the other containing 0.5 µl protein solution and 0.8 µl well solution. The wells contained 35 µl well solution and were stored at room temperature.

### 2.2. Data collection and processing

Protein crystals of diffraction quality were mounted with a nylon loop on a MicroRT-compatible loop holder and sealed with capillary tubing. The capillary tube tip contained 5 µl mother liquor and was sealed at the base with vacuum grease. Capillary sealing of the crystals and data collection were completed at room temperature. X-ray diffraction was collected on-site using a Rigaku XtaLAB Synergy-S diffractometer with a HyPix-6000HE detector. The raw data were processed using *CrysAlis^Pro^* and were scaled and merged with *AIMLESS*. The crystal structures were determined by molecular replacement in *Phaser* (McCoy *et al.*, 2007 ▸) using phosphate-bound ecGLK (PDB entry 9duc) as the starting model for both the K214Q and K216Q variant structures. Structural refinement was completed using *AutoBuild* for initial automated model building following molecular replacement (Terwilliger *et al.*, 2008 ▸), and *phenix.refine* was used to optimize the atomic models against experimental diffraction data by refining coordinates, atomic displacement parameters and occupancies, ensuring that the geometry of the model remains physically realistic while improving its fit to the map (Afonine *et al.*, 2012 ▸). The structures were manually adjusted in *WinCoot* for validation and refinement (Emsley *et al.*, 2010 ▸), and were then deposited in the PDB. The PDB codes for the ecGLK K214Q and K216Q variant structures are 9yrs and 9pyu, respectively. Table 1 ▸ lists diffraction data-collection and structure-refinement statistics.

## 3. Results and discussion

### 3.1. Phosphate-bound wild-type ecGLK defines the open and closed conformations upon glucose binding

**Figure 1:**
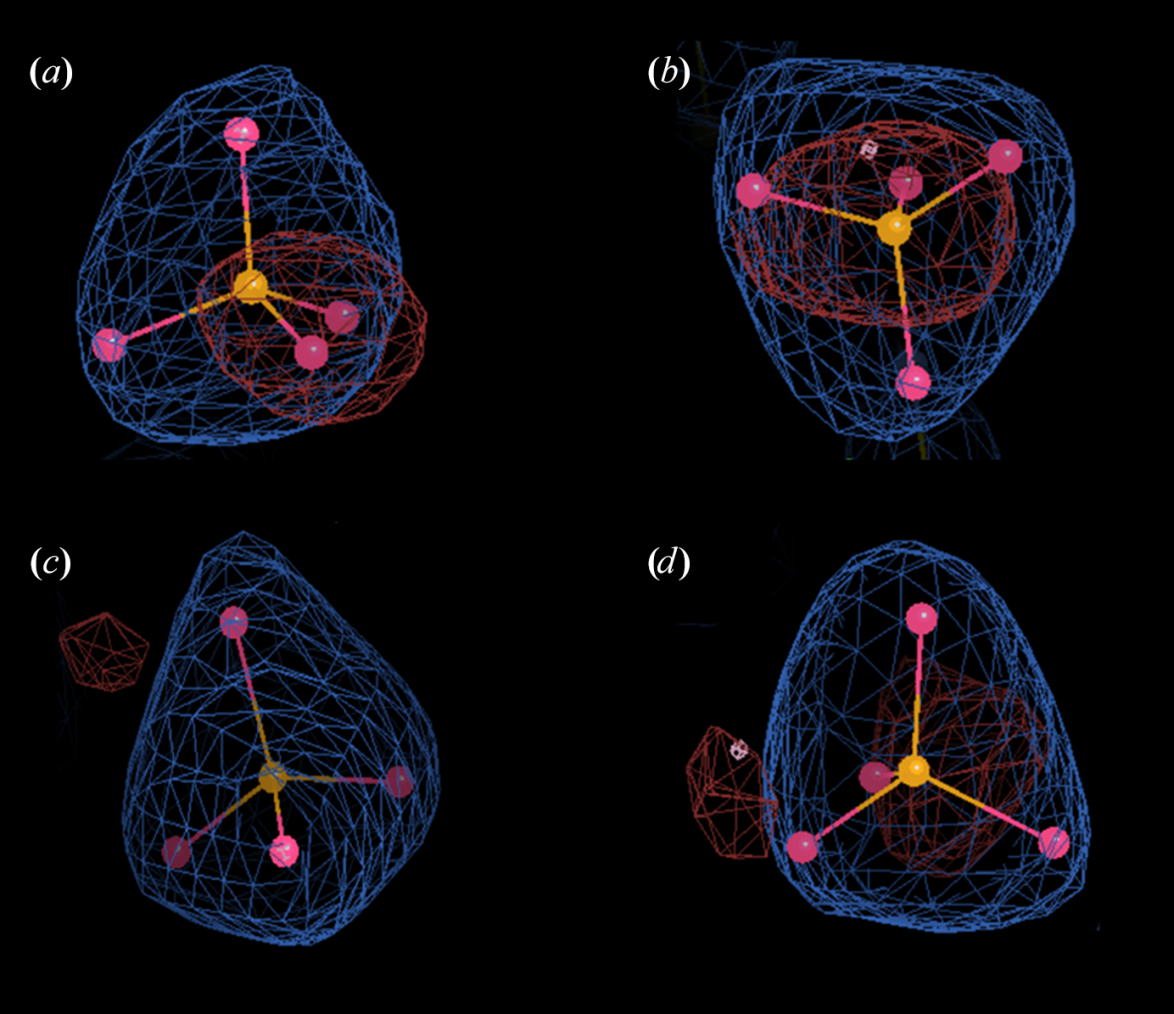
Electron densities for phosphate in ecGLK K214Q and ecGLK K216Q models. The blue mesh density surrounding the phosphate-occupied sites represents the 2*F*_o_ − *F*_c_ weighted electron-density map and the *F*_o_ − *F*_c_ difference electron-density map. (*a*, *b*) Phosphate in the model of ecGLK K214Q (PDB entry 9yrs); (*c*, *d*) phosphate in the model of ecGLK K216Q (PDB entry 9pyu).

To assess how the K214Q and K216Q mutations alter ligand-dependent conformational changes, the binding conformation of wild-type (WT) ecGLK was used as a reference framework. The structures of phosphate-bound ecGLK (PDB entry 9duc) and the phosphate-bound glucose–ecGLK complex (PDB entry 9dvz) were compared. As reported previously, the phosphate anion stabilizes the open conformation of the anion-binding pocket, which is formed by the His160, His183, Ser185, Arg188 and Arg286 residues (Andrews *et al.*, 2025 ▸). Consistent with this role, phosphate binding preserves a nearly identical geometry between the models, with deviations of less than 0.3 Å (Fig. 1 ▸).

**Figure 2:**
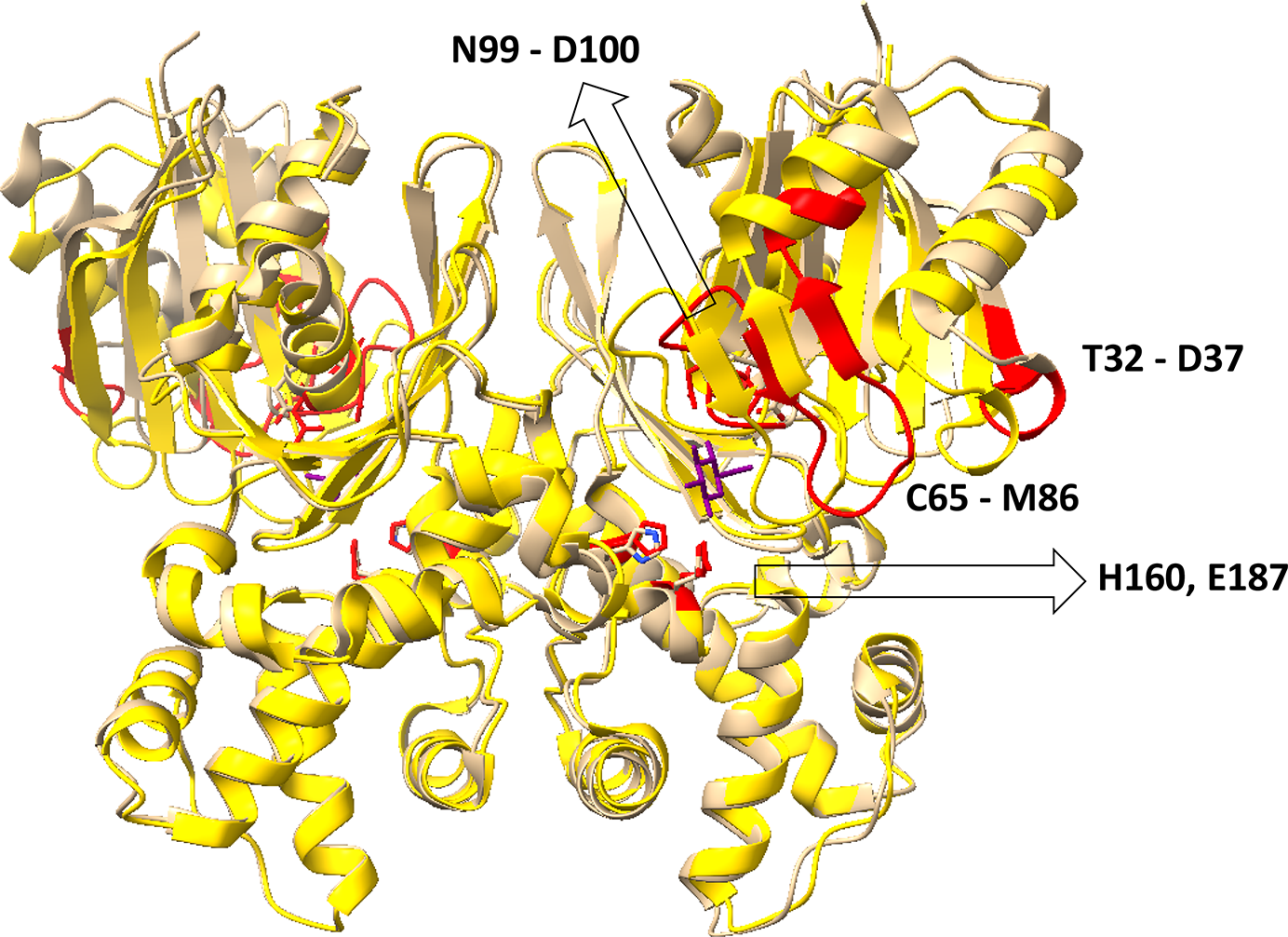
Comparison of the structures of phosphate-bound WT ecGLK (PDB entry 9duc, tan) and phosphate-bound ecGLK–glucose complex (PDB entry 9dvz, yellow). Sections with the largest observed differences are highlighted in red. Glucose is in purple.

In contrast, glucose binding induces coordinated movements of domains and loops. Notably, residues within the loop region (residues 32–90) shift by approximately 1.0–2.0 Å, driving closure of the large and small domains. This closure repositions key glucose-binding residues: Asn99 moves inwards towards glucose O2 by ∼0.8 Å, Asp100 shifts towards O3 by ∼1.0 Å, His160 rotates to engage O4 and Glu187 moves inwards to stabilize O5 and O6. Collectively, these rearrangements assemble the closed, catalytically competent state of ecGLK (Fig. 2 ▸).

### 3.2. The structure of the K214Q variant revealed intact phosphate binding but impaired organization of the glucose-binding pocket

To determine how the K214Q mutation alters phosphate binding and glucose-induced conformational transitions, the crystal structure of phosphate-bound ecGLK K214Q variant was determined and compared with the WT enzyme. Crystallization of the phosphate-bound ecGLK K214Q variant was achieved using a solution consisting of 100 m*M* MES–sodium hydroxide pH 5.5, 100 m*M* ammonium citrate dibasic, 20%(*w*/*v*) PEG 3350, 5%(*v*/*v*) 2-propanol. The structure, solved from crystals in space group *P*3_2_21 by molecular replacement, was refined to an *R* factor of 0.140 (*R*_free_ = 0.190) at a resolution of 2.70 Å.

The phosphate-bound K214Q ecGLK structure forms a dimer, with each subunit adopting the characteristic α/β architecture observed in WT ecGLK. The small domain comprises residues 2–110 and 300–321, while the large domain spans residues 111–299. The small domain contains β-strands β1–β4 and β7, as well as α-helices α1–α3 and α11, with helix α3 (residues 100–110) connecting the small and large domains. The large domain includes β8–β13 and α4–α10. Dimerization is mediated by helix α4 and adjacent loops, the C-terminal portion of α7, β10 and the loop connecting β10 and β11. The phosphate-binding pocket is located at the interface between the small and large domains, outside the α4–α10 region.

**Figure 3:**
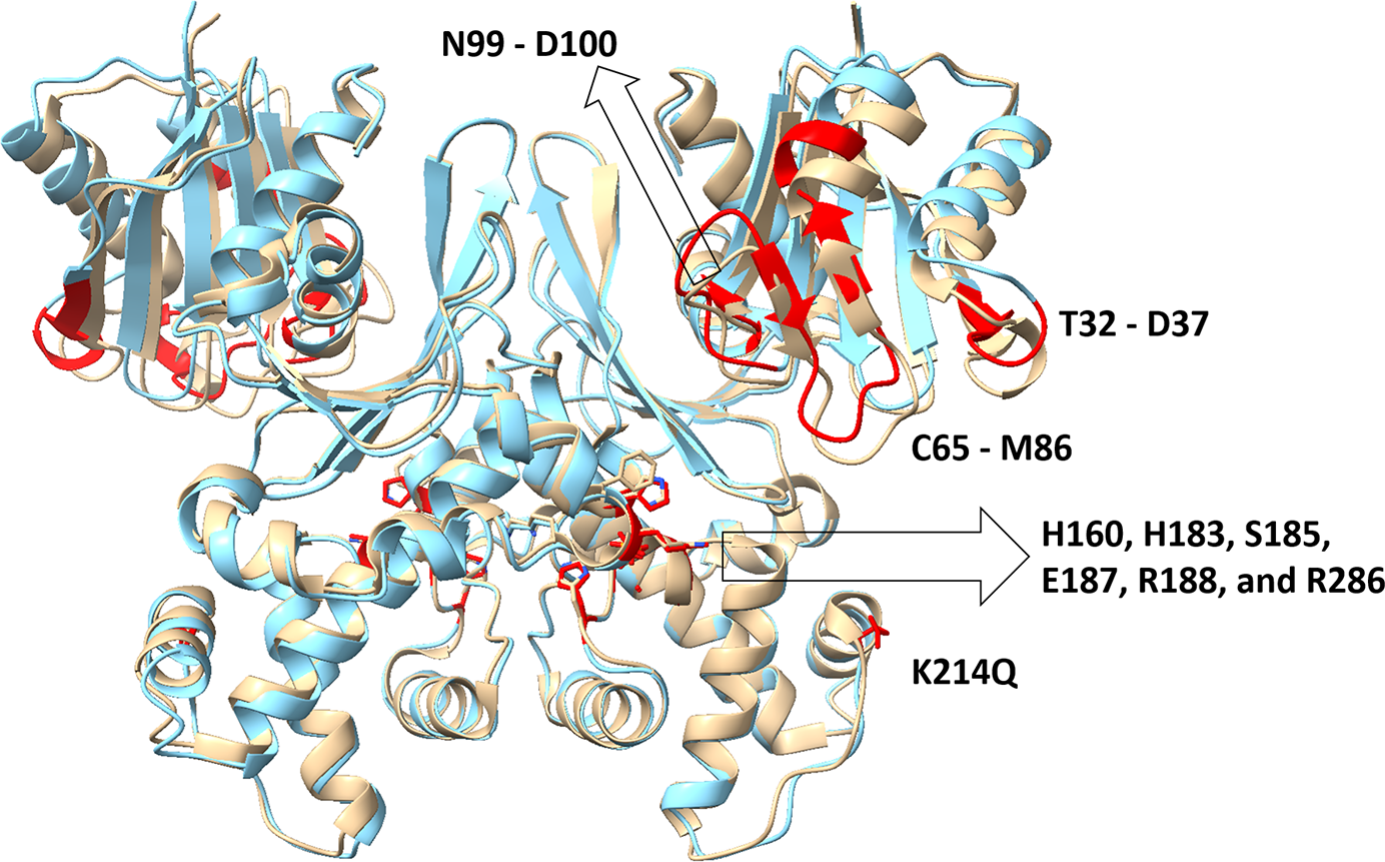
Comparison of the structures of phosphate-bound WT ecGLK (PDB entry 9duc, tan) and phosphate-bound ecGLK K214Q variant (PDB entry 9yrs, cyan). Sections with the largest observed differences are highlighted in red.

**Figure 4:**
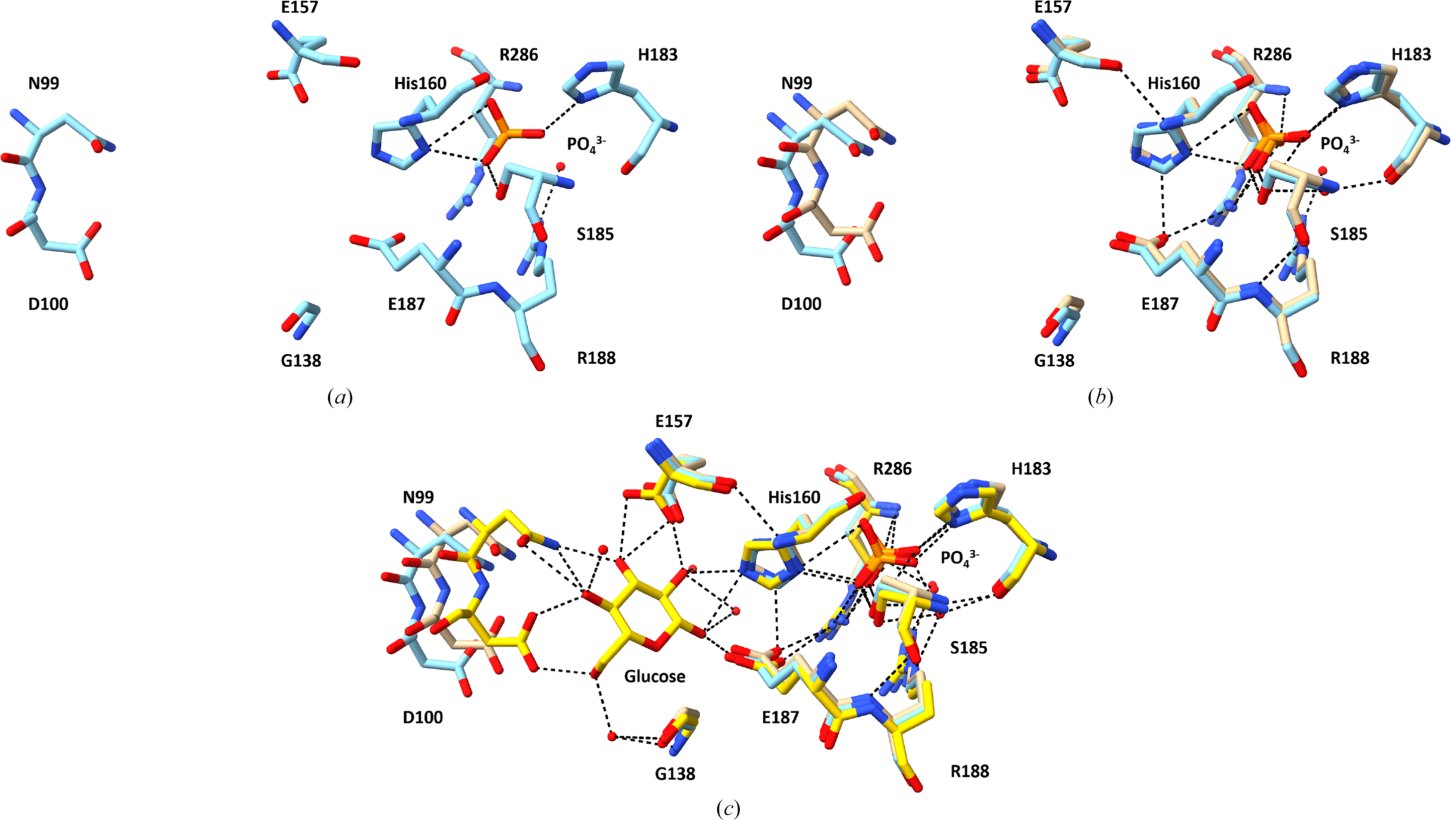
The structure of the glucose- and phosphate-binding sites of ecGLK K214Q. (*a*) Amino-acid residues in the glucose- and phosphate-binding sites of ecGLK K214Q (PDB entry 9yrs, cyan). (*b*) Superimposition of the residues in the glucose- and phosphate-binding sites of phosphate-bound WT ecGLK (PDB entry 9duc, tan) and phosphate-bound ecGLK K214Q (PDB entry 9yrs, cyan). (*c*) Superimposition of the residues in the glucose- and phosphate-binding sites of phosphate-bound WT ecGLK (PDB entry 9duc, tan), phosphate-bound ecGLK K214Q (PDB entry 9yrs, cyan) and phosphate-bound glucose–WT ecGLK complex (PDB entry 9dvz, yellow).

Superimposition of phosphate-bound ecGLK K214Q variant (PDB entry 9yrs) with phosphate-bound WT ecGLK (PDB entry 9duc) revealed localized but significant conformational differences (Fig. 3 ▸). In WT ecGLK, the phosphate group is stabilized by a well defined hydrogen-bonding network. His183 donates a hydrogen bond from ND1 to the O4 atom of phosphate, while Ser185 OG coordinates both O1 and O4. Arg188 NH2 interacts with a water molecule that bridges to phosphate O1, further reinforcing substrate binding (Fig. 4 ▸).

These interactions are reorganized in the K214Q variant. In chain *A* His160 ND1 forms new hydrogen bonds with phosphate O2 and O3, and Ser185 OG now interacts with O3. Additional stabilization arises from a rearranged hydrogen-bond network involving His160 NE2 and Glu187 OE1, as well as multiple water-mediated contacts contributed by His183, Ser185, Arg188 and Glu187. In chain *B*, phosphate binding remains anchored by conserved interactions with Arg286, which forms multiple direct hydrogen bonds with phosphate in both WT and K214Q ecGLK. However, a WT-specific hydrogen bond between Arg286 NH2 and Glu187 OE1 is lost in the mutant and is replaced by a new interaction between Lys284 O and phosphate O2, suggesting compensatory remodeling of the binding environment.

Despite this extensive reorganization, the overall phosphate clamp remains intact in the K214Q variant. In contrast, residues involved in glucose recognition exhibit pronounced deviations from the closed-state geometry (Fig. 4 ▸*c*). Asn99 is displaced outwards by 0.8–1.0 Å, likely impairing its inter­action with glucose O2. Asp100 shifts outwards by 0.9–1.2 Å, eliminating its ability to engage glucose O3. His160 moves further towards the phosphate-binding pocket, while Glu187 is displaced by ∼0.7 Å, disrupting potential interactions with glucose O5 and O6. These changes correlate with increased disorder in hinge and loop regions, particularly residues 32–37 and 65–86, which display r.m.s.d. values of 1.0–1.5 Å.

Together, these results indicate that while the K214Q mutation preserves phosphate binding through adaptive hydrogen-bond rearrangements, it disrupts the conformational transitions required to assemble the closed, glucose-bound catalytic state, thereby hindering efficient glucose recognition.

### 3.3. The structure of the K216Q variant showed modest perturbations and the retention of partial glucose-binding competency

To evaluate how the K216Q mutation affects phosphate binding and glucose-induced conformational changes, the crystal structure of the phosphate-bound ecGLK K216Q variant was determined and compared with WT ecGLK. Crystals of phosphate-bound ecGLK K216Q were obtained using a solution consisting of 100 m*M* MES–sodium hydroxide pH 5.5, 100 m*M* ammonium citrate dibasic, 20%(*w*/*v*) PEG 3350, 5%(*v*/*v*) 2-propanol. The structure was solved by molecular replacement in space group *P*3_1_21 and refined to an *R* factor of 0.1780 (*R*_free_ = 0.225) at a resolution of 2.44 Å. The model of the K216Q variant has a better resolution but higher *R* factors than that of the K214Q variant, which could be caused by a lower signal-to-noise ratio from unmodeled data noise or structural disorder. We also noted that the model of the K216Q variant has fewer water molecules than that of the K214Q variant but has a better resolution, possibly because only well ordered water molecules with low *B* factors were modeled to ensure high confidence.

**Figure 5:**
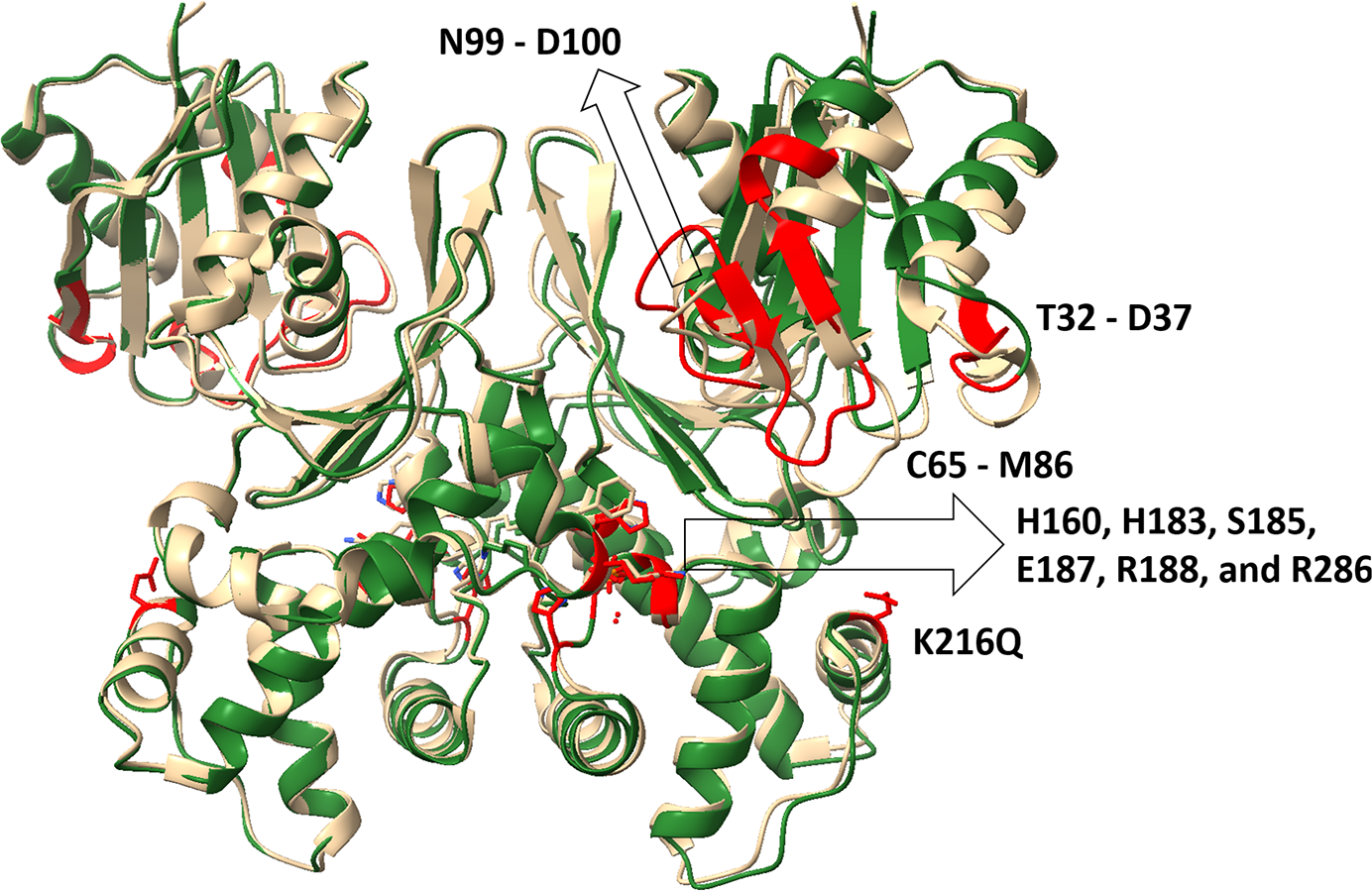
Comparison of the structures of phosphate-bound WT ecGLK (PDB entry 9duc, tan) and phosphate-bound ecGLK K216Q (PDB entry 9pyu, green). Sections with the largest observed differences are highlighted in red.

The overall architecture of the small and large domains, including the arrangement of α-helices and β-sheets, closely resembles that observed in WT ecGLK. Nevertheless, localized deviations are evident, reflecting mutation-induced conformational adjustments (Fig. 5 ▸).

**Figure 6:**
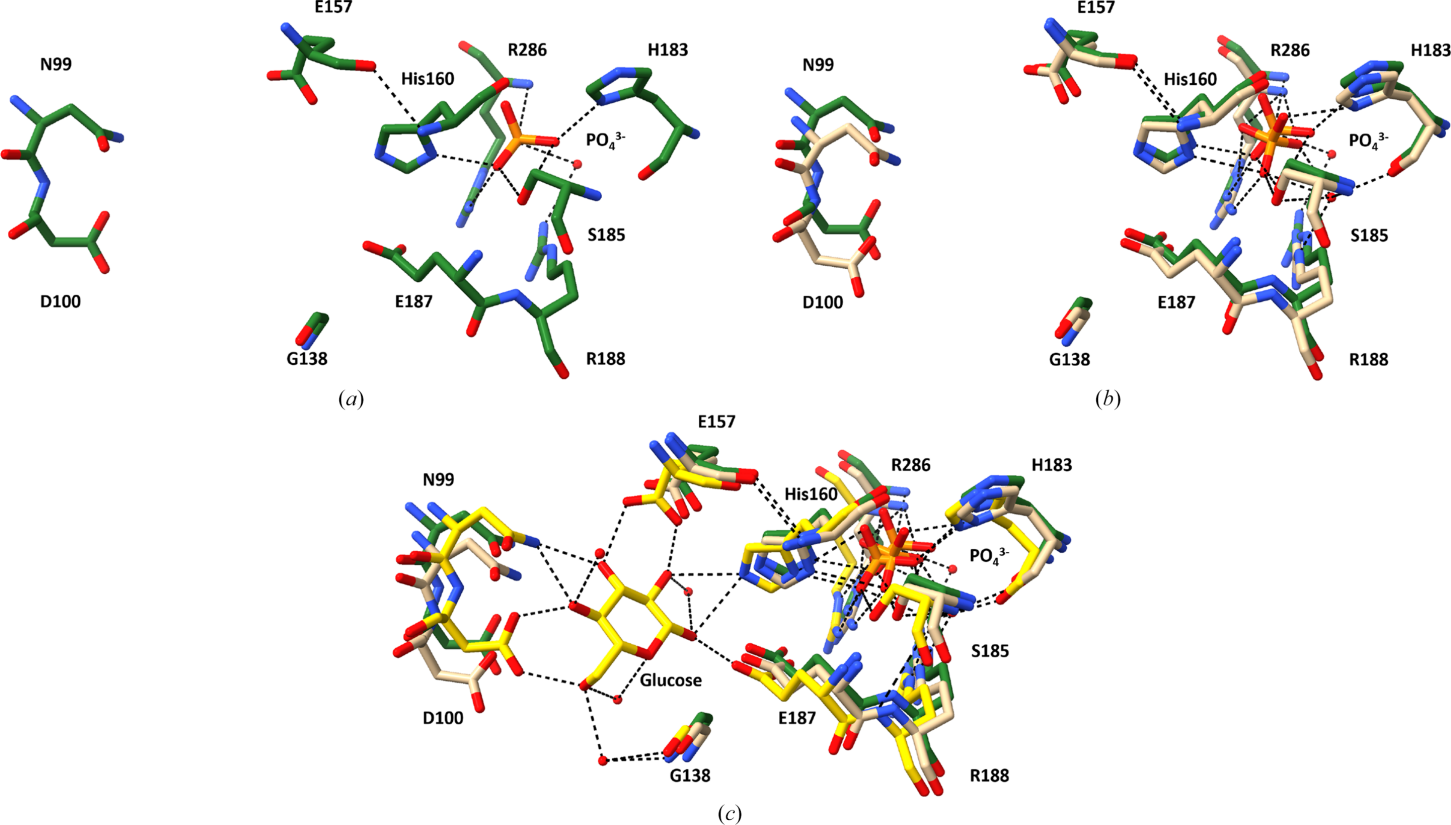
The structure of the glucose- and phosphate-binding sites of ecGLK K216Q. (*a*) Amino-acid residues in the glucose- and phosphate-binding sites of ecGLK K216Q (PDB entry 9pyu, green). (*b*) Superimposition of the residues in the glucose- and phosphate-binding sites of phosphate-bound WT ecGLK (PDB entry 9duc, tan) and phosphate-bound ecGLK K216Q (PDB entry 9pyu, green). (*c*) Superimposition of the residues in the glucose- and phosphate-binding sites of phosphate-bound WT ecGLK (PDB entry 9duc, tan), phosphate-bound ecGLK K216Q (PDB entry 9pyu, green) and phosphate-bound glucose–WT ecGLK complex (PDB entry 9dvz, yellow).

Analysis of the phosphate-binding site revealed a reorganization of hydrogen-bonding interactions in both subunits (Fig. 6 ▸). In WT ecGLK, Arg286 stabilizes phosphate by donating hydrogen bonds from its NH2 group to phosphate O4 and from its backbone nitrogen to phosphate O3. These interactions are altered in the K216Q variant. In chain *A*, Arg286 instead forms hydrogen bonds from its NE and backbone nitrogen to phosphate O1, with additional contacts from NH2 and NE to O3, indicating a redistribution rather than a loss of stabilizing interactions.

In chain *B*, WT ecGLK displays a conserved hydrogen-bonding network in which His160 ND1 interacts with phosphate O4, Ser185 OG coordinates O4, and a water molecule bridges to phosphate O3. These interactions are absent in the K216Q structure. Instead, the mutant exhibits a revised network in which Ser185 and Arg188 engage water molecules, Ser185 OG and N form water-mediated contacts, and His183 O contributes to stabilization through solvent interactions. Additional hydrogen bonds involving His183 ND1 and Ser185 OG to phosphate O1, as well as from Arg188 NH2 to water, further illustrate the altered hydrogen-bonding strategy.

In contrast to the K214Q mutation, the K216Q mutation exerts only modest effects on the glucose-binding pocket (Fig. 6 ▸*c*). Asn99 is displaced outwards by 0.4–0.6 Å, weakening but not abolishing its interaction with glucose O2, while Asp100 shifts by ∼0.5 Å, slightly diminishing its ability to engage glucose O3. His160 adopts a rotameric state intermediate between the open and closed conformations, consistent with readiness for domain closure. Glu187 is displaced outwards by only 0.3–0.5 Å, suggesting reduced but retained capacity to stabilize glucose O5 and O6.

Overall, these observations indicate that while the K216Q substitution remodels the hydrogen-bonding network at the phosphate-binding site, it largely preserves the structural framework required for glucose binding and domain closure, distinguishing it from the more disruptive effects observed for the K214Q mutation.

### 3.4. Lys214 and Lys216 could have different structural roles

**Figure 7:**
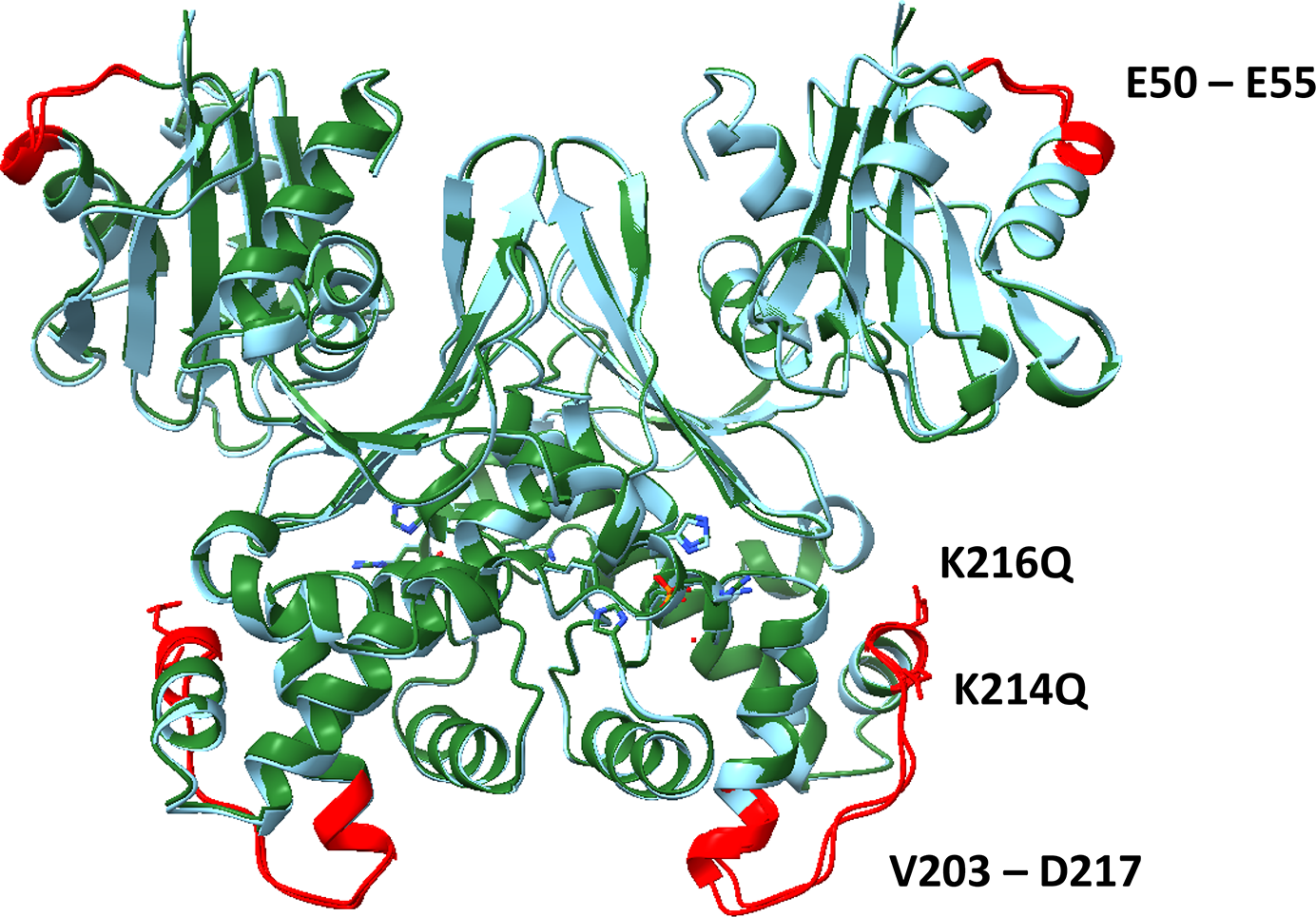
Comparison of the structures of phosphate-bound ecGLK K214Q (PDB entry 9yrs, cyan) and phosphate-bound ecGLK K216Q (PDB entry 9pyu, green). Sections with the largest observed differences are highlighted in red.

Although Lys214 and Lys216 reside on the same α-helix (residues 214–224), their substitution with glutamine produces different structural consequences (Fig. 7 ▸). In the K214Q variant, the glucose-binding residues Asn99, Asp100, His160 and Glu187 are displaced outwards by 0.6–1.2 Å, whereas the corresponding shifts in the K216Q variant are smaller, ranging from 0.3 to 0.6 Å. Consistent with these differences, loop disorder is more pronounced in the K214Q variant (r.m.s.d. 1.0–1.5 Å) than in the K216Q variant (r.m.s.d. 0.7–0.9 Å), indicating that Lys214 plays a critical role in maintaining the active-site geometry and enabling loop-mediated stabilization of domain closure.

It should be noted that glutamine is smaller than authentically acetylated lysine. Accordingly, the structural effects of acetylation at Lys214 or Lys216 are likely to be more pronounced than those observed for glutamine substitutions, consistent with our previous kinetic analyses (Fatema *et al.*, 2024 ▸).

Together, the WT and variant structures support a two-step mechanism for ligand recognition by ecGLK. Phosphate binding at the anion-binding site stabilizes the open conformation, whereas glucose binding induces inward movements of key residues and loops that drive domain closure. In the K214Q variant, these transitions are impaired: glucose-binding residues remain in a conformation more open than the WT open state, and increased loop disorder dampens the propagation of closure motions (Fig. 4 ▸*c*). By contrast, the K216Q variant remains partially competent, retaining sufficient residue alignment to support near-wild-type catalytic activity despite increased flexibility (Fig. 6 ▸*c*).

The distinct structural consequences of the K214Q and K216Q mutations underscore the distributed nature of conformational regulation within ecGLK. Lys214 serves as a critical stabilizing element for glucose-binding residues, whereas Lys216 primarily modulates the flexibility of hinge and loop regions that facilitate glucose-induced closure. Although both variants preserve the phosphate-binding geometry, the K214Q mutation leads to substantial backbone rearrangements and local disorder that disrupt glucose stabilization, while the K216Q mutation results in only modest perturbations. Collectively, these findings demonstrate that adjacent lysine residues can fulfill distinct structural roles and highlight the importance of precise helix packing in sustaining the conformational transitions required for efficient gluco­kinase function.

It is unclear why the K214Q mutation causes the significant structural variation from our models. One possible reason could be related to ATP binding. Based on the predicted ATP-binding site from the previous study (Lunin *et al.*, 2004 ▸), Lys214 is close to the ATP-binding site. Acetylation of the lysine residue neutralizes the positive charge, thus causing conformational changes. Further investigation, *i.e.* co-crystallization of ecGLK with ATP, is ongoing.

## Supplementary Material

PDB reference: *Escherichia coli* glucokinase, K214Q variant, 9yrs

PDB reference: K216Q variant, 9pyu

## Figures and Tables

**Table 1:** Crystallographic data and refinement statistics for X-ray structures Values in parentheses are for the highest resolution shell.

	ecGLK K214Q	ecGLK K216Q
Data collection		
Space group	*P*3_2_21	*P*3_2_21
*a*, *b*, *c* (Å)	84.97, 84.97, 183.31	85.09, 85.09, 183.32
α, β, γ (°)	90.00, 90.00, 120.00	90.00, 90.00, 120.00
Radiation source	Rigaku PhotonJet-S	Rigaku PhotonJet-S
Wavelength (Å)	1.5418	1.5418
Resolution limits (Å)	30.55–2.70 (2.83–2.70)	28.72–2.44 (2.54–2.44)
*R*_merge_† (within *I*^+^/*I*^−^)	0.139 (0.644)	0.356 (5.373)
*R*_merge_† (all *I*^+^ and *I*^−^)	0.146 (0.682)	0.372 (5.557)
*R*_meas_‡ (within *I*^+^/*I*^−^)	0.157 (0.723)	0.397 (5.939)
*R*_meas_‡ (all *I*^+^ and *I*^−^)	0.156 (0.722)	0.393 (5.856)
*R*_p.i.m._§ (within *I*^+^/*I*^−^)	0.072 (0.324)	0.171 (2.467)
*R*_p.i.m._§ (all *I*^+^ and *I*^−^)	0.052 (0.234)	0.126 (1.824)
*R*_merge_ in top intensity bin	0.050	0.081
No. of observations	192472 (27023)	279237 (32649)
No of possible unique observations	21813 (2874)	29403 (3239)
No. of unique observations	21791 (2874)	29374 (3239)
Mean *I*/σ(*I*)	11.6 (3.5)	5.6 (0.6)
CC_1/2_	0.994 (0.891)	0.990 (0.211)
Completeness (%)	99.9 (100)	99.9 (100)
Multiplicity	8.8 (9.4)	9.5 (10.1)
Mean χ^2^	0.97 (0.87)	0.99 (0.98)
Refinement statistics		
Resolution (Å)	30.55–2.70	28.72–2.44
Protein residues	640	640
Solvent molecules	119	89
Phosphate molecules	2	2
*R*_free_	0.190	0.225
*R*_work_	0.140	0.178
R.m.s.d.		
Bond lengths (Å)	0.004	0.002
Angles (°)	0.629	0.418
Residues in Ramachandran plot	640	640
Most favored regions (%)	94.81	93.72
Allowed regions (%)	5.19	6.28
Mean *B* factors (Å^2^)		
Protein	49.3	57.4
Phosphate	36.6	48.24
Wilson *B* factor (Å^2^)	28.9	13.4

† *R*_merge_, also known as *R*_sym_, is a discrepancy index used to measure the equivalence or internal consistency among intensities of equivalent reflections in X-ray crystallo­graphy.

‡ *R*_meas_ is a discrepancy index introduced to account for the dependence of *R*_merge_ (*R*_sym_) on the multiplicity, which is the number of equivalent reflections.

§ *R*_p.i.m._, or the precision-indicating merging *R* factor, is a discrepancy index introduced to further refine the dependence of *R*_meas_ on multiplicity.

## Data Availability

All data created during this research are openly available from the Protein Data Bank under the provided accession codes. This study includes the re-analysis of existing data, which are openly available at the locations cited in the references section.
